# Biological and Chemo-Physical Features of Denture Resins

**DOI:** 10.3390/ma13153350

**Published:** 2020-07-28

**Authors:** Gabriele Cervino, Marco Cicciù, Alan Scott Herford, Antonino Germanà, Luca Fiorillo

**Affiliations:** 1 Department of Biomedical and Dental Sciences, Morphological and Functional Images, University of Messina, Policlinico G. Martino, Via Consolare Valeria, 98100 Messina, Italy; gcervino@unime.it (G.C.); lfiorillo@unime.it (L.F.); 2 Department of Maxillofacial Surgery, Loma Linda University, Loma Linda, CA 92354, USA; aherford@llu.edu; 3 Department of Veterinary Sciences, University of Messina, 98122 Messina, Italy; agermana@unime.it

**Keywords:** dental materials, resin, acrylic, biomechanics, dentistry, dentures, biofilm, bacteria, dental technician

## Abstract

In the dental field, the study of materials has always been the basis of the clinical practice. Over the years, with the evolution of materials, it has been possible to produce safe and predictable prosthetic devices, with ever better aesthetic features, biocompatibility and patient satisfaction. This review briefly analyzes the features of dental resin materials to underline the biological, microbiological and chemo-physical characteristics. The main aim of prosthodontics is to rehabilitate patients and therefore improve their quality of life. Dental resins are the main materials used for the production of dentures. Once solidified, these polymers have different mechanical or surface characteristics. The results of the literature on these characteristics were analyzed and some new brand dental resins, known as modern resin, were subsequently evaluated. The new materials are undoubtedly a step forward in the creation of dental prostheses, and also in all subsequent maintenance phases. This review shows how changing the chemical structure of the resins could have microbiological influences on the growth and management of the biofilm, and also physical influences in terms of its mechanical characteristics. The development of new materials is a constant goal in dentistry in order to obtain increasingly predictable rehabilitations.

## 1. Introduction

### 1.1. Background

Mobile dental prostheses aim to rehabilitate the oral functions of patients suffering from partial or total edentulism by replacing natural teeth with artificial dental elements and tissues. Some types of prosthetic interventions could be used to correct functional anomalies as well as aesthetics of shape, color or position of natural teeth [1,2,3,4]. For this to happen, dental technology has relied on the field of dental materials for years to obtain better performing, compatible and long-lasting materials [5,6,7,8].

Artificial or synthetic resin generally refers to a viscous material, similar in appearance to vegetable resin and capable of hardening. It is generally a wide class of different and complex polymers, which could be obtained with a large variety of methods and raw materials. Among the most common synthetic resins are phenolic resins, acrylic resins, epoxy resins, unsaturated polyester resins (UPR), vinyl ester resins (VE), thermoplastic resins, thermosetting resins and elastomers. Acrylic resins (polyacrylates) are obtained by polymerization of acrylic monomers, mainly acrylic acid and acrylic or methacrylic esters [9,10,11,12]. The comonomer mixture is optimized to obtain copolymers with particular characteristics, such as flame resistance, elasticity, cross-linkability, antistatic behavior etc [13,14]. The main applications include paints for construction, coating of metals, adhesives and sealants, coating of paper, fabrics and leather or even in the dental field as an aesthetic material for the construction of prostheses [15,16,17]. Synthetic resins are particular materials with physical, chemical and aesthetic characteristics that allow them to be widely used in dental technology. They have the fundamental characteristic of being able to take on the most varied forms in certain conditions of temperature and pressure. Chemically they originate from well-defined compounds (polymers), which, with the intervention of suitable catalysts (monomers), give rise to the chemical reaction called polymerization which allows one to obtain a prosthesis with adequate characteristics [18,19,20,21,22].

### 1.2. Aim

The aim of this narrative review is to evaluate what the current chemical–physical characteristics of the dental resins that are used for total prostheses, and therefore consequently their clinical features. Surface outcomes, biocompatibility or ability to give inflammatory reactions, plaque’s ability to adhere, and biomechanical features will certainly be included among the outcomes taken into consideration. This material has been minimally explored in the literature and systematic reviews on it are still few in number and the objectives are also different, for example, some evaluate only new materials such as polyamide [23,24].

The main questions of this systematic review are the following:

- Does dental resin material feature influence predictability of the rehabilitation in patients who have dentures? Are dental resin material characteristics influenced by composition in denture?

## 2. Materials and Methods

### 2.1. Protocol and Registration 

The following systematic review was conducted in accordance with the PRISMA protocols (preferred reporting items for systematic reviews and meta-analyses) [25,26,27]. The following systematic review was also recorded on the PROSPERO (International Prospective Register of Systematic Reviews) website and is accessible with the protocol number 190790 and date 06/06/2020. In addition, the PICO protocol was used to formulate the main question of this systematic review.

### 2.2. Eligibility Criteria 

The following inclusion and exclusion criteria were used to carry out this systematic review.

Inclusion criteria:

- Scientific articles concerning the dental materials of removable prostheses.
- Scientific articles containing information on dental resins.
- Scientific articles concerning chemical–physical and biological interface information on dental acrylic resins.

Exclusion criteria:

- Resins used for other purposes or in other areas of medicine.
- Items not accessible, with missing or incomplete dates.
- Articles not in English.
- Short articles, theses, or letters.

### 2.3. Information Sources 

The sources of information used to search for results in this systematic review include different scientific search engines: Pubmed, Embase, Scopus and MDPI (Multidisciplinary Digital Publishing Institute). The latest research was carried out in June 2020.

### 2.4. Search 

The following keywords were searched in the scientific search sources mentioned in Section 2.3, with the aim of obtaining the highest possible number of results:

“Denture resin” AND “Acrylic”

### 2.5. Study Selection 

After careful electronic selection, manual studies related to the topic were selected. Subjects of great scientific interest and research of recent publication were selected, according to the eligibility criteria.

### 2.6. Data Collection Process 

The first phase of the research consisted of the selection of titles, which allowed us to make a first screening of the manuscript, eliminating those not concerning this research. Finally, the full text of all studies was obtained and according to the expected inclusion/exclusion criteria, articles were selected and included in the present review.

### 2.7. Data Items 

Dental resins provide mechanical characteristics and interaction with specific tissues so that they can be used. Synthetic resin requirements:

- Adequate mechanical and chemical characteristics: they should have high elasticity and resistance as they should bear the weight of the chewing load or the stresses of the buccal liquids.
- High chemical stability.
- Good aesthetic characteristics: the color and translucency should be similar to natural tissue (it is important that the color is maintained over time).
- Insolubility in buccal fluids and absorption of these in the least amount possible.
- Low density, particularly total prostheses should be light and reproduce at the same time all the morphological details.
- High softening temperature, such as not to generate deformations of the prosthesis in the oral cavity.
- Absence of taste, smell and of irritative and allergic phenomena [28,29,30,31].

Currently, the most used synthetic resins are acrylic resins based on polymethylmethacrylate: this is an acrylic resin obtained by the polymerization of methyl methacrylate. Methyl methacrylate is the methyl ester of methacrylic acid. Synthetic resins could be subdivided into two groups: heat-cured and cold-cured. The former needs a certain amount of heat to make the polymerization occur and therefore obtain all the necessary requirements for a correct prosthetic reconstruction. The cold-cured agents do not require external heating as the polymerization occurs spontaneously at room temperature (the composition of the powder and the liquid are the same as the heat-cured ones, but the addition of a chemical activator gives rise to polymerization even at room temperature, as when present in the liquid, it mixes with the benzoyl peroxide in the powder as an initiator). The most appropriate proportion between the polymer (powder) and the monomer (liquid) is three parts to one by volume and two parts to one by weight. Polymer high percentage tends to lower the reaction time and the tendency of the resin to contract during polymerization; on the other hand, it is advisable to use an adequate quantity of monomer so that it can completely wet the polymer particles (in fact the polymer–monomer proportions may vary according to the size of the particles of the polymer powder). These resins could be used for:

- Synthetic resins for prosthetic bases: They are used in mobile prostheses for their characteristics, in fact their main component is polymethyl-methacrylate.
- Resins for relining of mobile prostheses: The soft tissues underlying the prosthetic bases tend to undergo changes in shape over time due to the slow reabsorption of the underlying bone tissue. It is therefore necessary to change the shape of the surface of the resin prosthesis that comes into contact with the mucosa to maintain adequate adhesion. For this operation, resins similar to the previous ones need to be used, but they need to be able to perfectly adapt to achieve the desired purpose.
- Resins for repair of mobile prostheses: Despite the constant stresses that prostheses undergo during normal chewing functions, relatively few fractures occur in the mouth; this is often due to too thin bases and too deep or acute frenum measurements.
- Artificial resins: These are similar in composition to those for prosthetic bases but contain a greater concentration of substances that increase their wear resistance and the weight of the chewing load (the part of the teeth that is fixed to the resin base, however, contains a lower amount of these substances in order to allow a correct union with the resin of the base itself).
- Resins for fixed prosthesis crowns and bridges: For this use, various types of resins are available that have a wide range of colors similar to natural teeth, heat-cured acrylic resins, thermopolymerizable vinyl-acrylic copolymers, modified acrylic resins, acrylic resins with reinforcing substances, and composite resins based on the Bowen monomer [32,33,34].

### 2.8. Risk of Bias

Risk of bias has been addressed according to [35,36,37]. In statistics, the terms bias, distortion or variance are used with reference to two concepts. A distorted sample is a statistical sample in which the probability of inclusion in the sample of individuals belonging to the population depends on the characteristics of the population under study (Table 1). 

### 2.9. Summary Measures 

A summary of the measures assessed in this review can be expressed as follows:

- Table 1
  ◦ Author and year—author and year of publication;
  ◦ Type of study—type of manuscript (article, Randomized Clinical Trials (RCT), review, etc.);
  ◦ Sample size and type—sample size and type of performed analysis (in vitro, in vivo, in silico etc);
  ◦ Intervention/method—type of group subdivision and features;
  ◦ Main outcomes—intervention on single specimens and type of evaluated outcomes;
  ◦ Main results—main results of the single study;
  ◦ Statistical analysis—statistical data regarding outcomes.
- Table 2
  In this table, the Risk of Bias in systemic Review (ROBIS) [35,36,37] method for risk of bias allocation was used.
- Table 3
  ◦ Biological features—only biological outcomes, host tissue or cell implications.
  ◦ Microbiological features—microbiological outcomes, bacteria, fungi or virus.
  ◦ Physical features—physical, mechanical, chemical properties.
  ◦ Other—other outcomes, in this case, only one result evaluated “patient acceptability”.

### 2.10. Synthesis of Results 

The data obtained from the individual results are summarized in the “Results” section. The synthesis of the results was conducted manually by the individual authors, independently. Once the titles and abstracts were screened, the individual authors extrapolated the results from the individual articles and they were compared at the end of the review process.

### 2.11. Additional Analysis

To give the reader readiness of what has been analyzed in this review, an examination was chosen to closely observe the microscopic surface characteristics of a cold-cured resin. The resin in question is a liquid powder resin, composed of:

- Liquid: methacrylate, tetramethylene, dimethacrylate.
- Powder: dibenzoyl peroxide, methyl methacrylate (does not contain cadmium).

**Figure 1:**
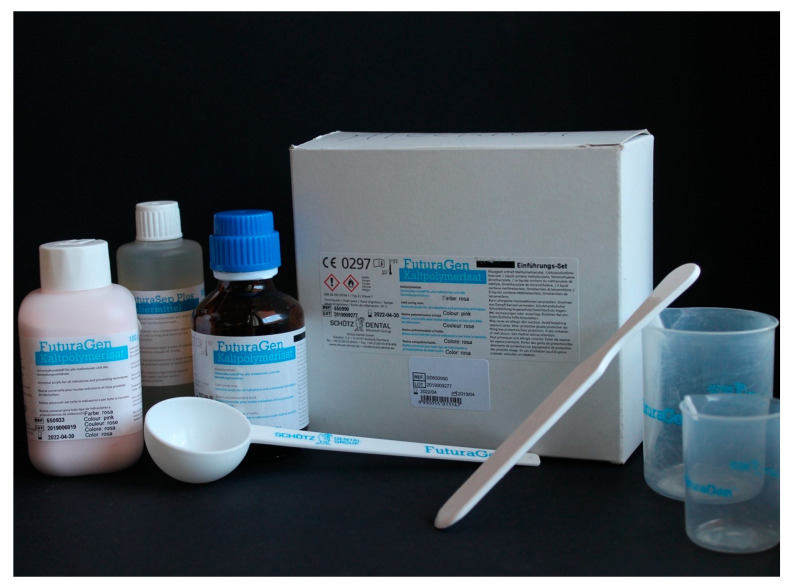
Sample of dental pink resin kit, containing (from left to right) resin powder, insulating liquid, resin liquid, a measuring spoon, a spatula, liquid and powder bakers.

A common resin used in dentistry (FuturaGen^®^ Schutz Dental GmbH, Rosbach, Germany), pink in color, is presented in Figure 1, where it is possible to observe how this resin presents itself to the clinician. The resin surface and fractured surface were observed with a stereomicroscope (Leica^®^ M125 C). Once the resin was mixed according to the manufacturer’s instructions, two sample were created (4 × 2 × 1 cm); these were subsequently fractured into two equal parts, and the surface was observed. Images were modified and optimize by applying mac Os photo^®^.

## 3. Results

### 3.1. Study Selection 

**Figure 2:**
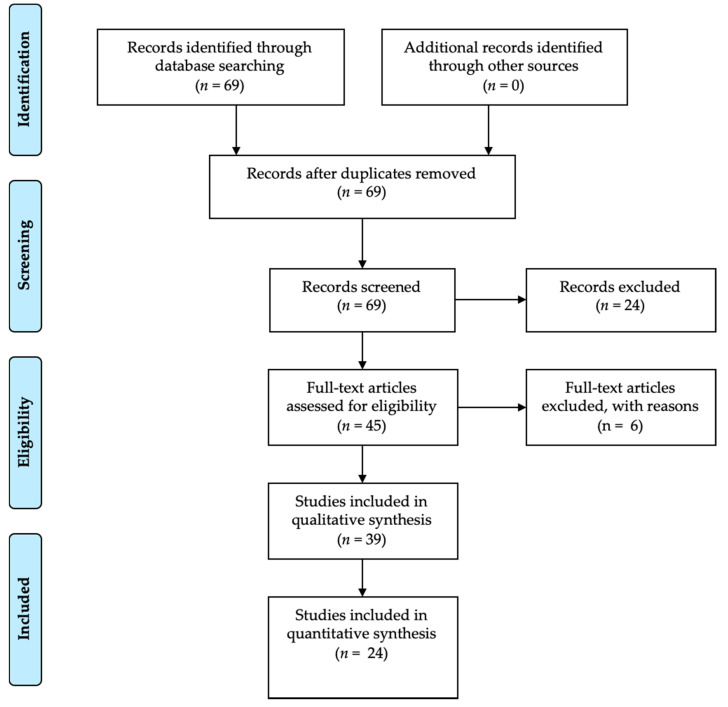
PRISMA flow chart.

A first search resulted in a total of 69 manuscripts. Subsequently, with the application of the inclusion and exclusion criteria according to the Materials and Methods Section, that is, limiting the results of the last 20 years, the number of results was reduced to 45. Subsequently, only the full texts (24) were evaluated (Figure 2).

### 3.2. Study Characteristics 

The main study features are reported in Table 1 according to Materials and Method Section. In Table 1, as specified in the previous paragraphs, it is possible to quickly observe the results obtained from the review. It is important to report in the Intervention/Method column how the groups present and the individual investigations are carried out, while in the subsequent columns, the main outcomes are reported, noting any further treatments on the resins and the results.

### 3.3. Risk of Bias

A risk of bias analysis was performed following in accordance with the described methods in the Materials and Methods Section; data are reported in Table 2.

### 3.4. Results of Individual Studies

All the results obtained from the analysis of the individual manuscripts are listed in Table 3 and divided according to the Materials and Methods Section.

### 3.5. Synthesis of Results 

#### 3.5.1. Microbiological Properties

Bacali et al. [38] evaluated some clinical and chemo-physical features of modified denture resins. The authors evaluated the feature of auto-polymerizing acrylic resin loaded with 1% and 2% of G-AgNp (graphene–Ag nanoparticles). They demonstrated how these resins could interact with and decrease cell viability on dysplastic oral keratinocytes and dental pulp stem cells. Pro-inflammatory molecules decreased in G-AgNp samples, demonstrating an antioxidant effect too. All samples, according to authors, demonstrated antibacterial properties against Gram-positive bacteria and the bactericide effect of Escherichia coli. From a mechanical point of view, these modified resins showed improved flexural strength. Alfaifi et al. [39] demonstrated how different concentrations of caffeine and nicotine could interact with the metabolic activity and biofilm formation of Candida albicans. They showed how 8 mg/mL of nicotine increased both metabolic activity and biofilm formation. Despite this, high caffeine concentration (16.00 and 32.00 mg/mL) could decrease the metabolic activity and biofilm formation of *C. albicans*. Akalin-Evren et al. [46] evaluated *C. albicans* adhesion on resin dentures reinforced with FRC fiber-reinforced composites. FRC architecture (woven or unidirectional) did not influence adhesion, nor differences between exposed dentures to saliva or distilled water. Fan et al. [51] used both light-cured and chemical-cured systems to synthesize AgNPs using different concentrations of Ag benzoate (AgBz). These concentrations were verified thought an electron microscopy, and Ag benzoate did not affect resin hardness. Antimicrobial resins released Ag+ ions in all samples, but they showed antimicrobial activity against Streptococcus mutans and showed an inhibition from 52.4% to 97.5%. Pesci-Bardon et al. [58] mixed Poly 202063, a quaternary ammonium compound polymer, with denture resin to evaluate its antimicrobial effect. They tested some resin discs with different inoculum volumes, concluding that the specimen provided antimicrobial and antifungal effect, with better results with low inoculum. 

#### 3.5.2. Biological Properties

Lee et al. [42] evaluated the cytotoxicity of different denture materials: polyamide, acrylic, polypropylene and a heat-polymerized acrylic resin as a control group. They obtained extracts from specimens of the denture materials under different condition (37 °C for 24 h, 70 °C for 24 h, and 121 °C for 1 h). The extracts were then diluted in distilled water and co-cultured for 24 h with immortalized human oral keratinocytes (IHOKs) or mouse fibroblast. Greater than 70% viability was detected under all test conditions.

Denture cleansers solution can affect denture properties. de Sousa Porta et al. [44] evaluated color, roughness change and biofilm formation with the use of sodium hypochlorite. The authors evaluated patient satisfaction after 90 days of the use of this solution. They evaluated the use of a 0.5% NaOCl solution for 3 min a day on an acrylic resin denture. They showed a significant reduction in biofilm formation with no roughness or color change, and with a better patient satisfaction after use. Monteiro et al. [49] showed silver distribution and release in antimicrobial base resin, with added silver colloidal nanoparticles. Acrylic resin was prepared in accordance with manufacturer’s instructions and silver nanoparticle suspension was added in different concentrations. After storing the dentures in deionized water for a time from 7 up to 120 days, they analyzed each solution. Silver was not detected in deionized water and they showed how silver dispersion was better at a lower silver concentration. Kim et al. [55] evaluated the biocompatibility of reinforced acrylic hybrid resin with polyhedraloligosilsesquioxane (POSS). POSS showed improved biocompatibility (measured by a metabolic assay, an agar overlay test, and a mutagenesis assay) and lower mutagenicity.

#### 3.5.3. Physical Properties

Al-Thobity et al. [40] evaluated the effect of cleansing solution on different denture resins (heat-polymerized, auto-polymerized, visible-light-polymerized). They evaluated the effect of distilled water as control, as well as Corega and Renew cleansing solutions. The only color change detected was in the visible-light-polymerized (VLP) resin treated with Corega and Renew. Surface roughness of all denture resin increased after immersion in Corega. Immersion in Renew significantly increased surface roughness only in the heat-polymerized (HP) and auto-polymerized (AP) specimens. A reduction in flexural strength was detected in the HP resin after immersion in Corega. Somkuwar et al. [41] evaluated the effect of multiwalled carbon nanotubes (MWCNTs) on PMMA (Poly(methyl methacrylate)) denture resin flexure strength. They demonstrated how microwave-cured denture resins have better flexure strength than the water bath-cured type, and that a 0.025% or 0.050% MWCNT weight could improve this physical property. 

Wagner et al. [43] evaluated dimensional stability of PMMA resin dentures after microwave irradiation. Denture bases were placed into a glass baker with 200mL of room-demineralized water and then exposed to 420W or 700W microwave radiation for 3 min. All dentures experienced a 1- or 2-mm dimensional change after each period of microwaving. Wang et al. [45] evaluated the effect of a multiwalled carbon nanotube on denture polymethyl methacrylate composite resins. In this in vitro study, they fabricated dentures with 0.5, 1 and 2 wt% of multiwalled carbon nanotubes. These resins were sonically mixed for 20 min and mechanical features were measured. The results suggested that the interfacial bonding between MWCNTs and PMMA was weak and in need of improvement. The addition of 0.5% and 1% MWCNTs improved the PMMA resin flexural strength and resilience, but the addition of 2% MWCNTs was not able to do so because of poor dispersion of the MWCNTs. Mansour et al. [47] evaluated the effect of mica on the flexural strength and microhardness of PMMA denture resin. They tested two mica, W200 with an average particle sizes (d50) of 131 µm and P66 with an average particle sizes (d50) of 30 µm. Dentures were fabricated according to the resin manufacturer’s instructions; different amounts of mica were added to each group. Mica seemed to give less flexural strength with high microhardness to dentures. So et al. [48] evaluated the effect of reinforcement by various concentrations of chopped E-glass fibers (0%, 1%, 2%, 3% and 5% by weight of resin powder) and post-curing microwave irradiation (800 W for 3 min) on the flexural strength of cold-cured acrylics. According to them, at room temperature and humidity for 1 day, the group with 3% and 5% fiber reinforcements obtained significant results compared to test group. They demonstrated how the effect that a water bath for several days could have on resin reinforcements. When the water storage time increased, the effect of the fiber remained, but the effect of microwave treatment vanished. Ladha et al. [50] evaluated the flexural strength of different reinforced PMMA dentures. They tested unreinforced resins, those reinforced with unidirectional stick glass fibers, woven stich net glass fibers and nylon fibers. After storing them in dry and wet conditions, they conducted a 3-point bending flexural test. Glass fiber improved flexural strength, and nylon fiber decreased it, even more than unreinforced acrylic resin. 

Zortuk et al. [52] evaluated the influence of different concentrations of fiber glass on resin surface roughness. After polishing the specimens, they evaluated and calculated the surface roughness through a profilometer. They observed significant differences between groups, with fiber glass groups presenting a higher surface roughness than the non-fiber group. Puri et al. [53] evaluated the effect of ethylene glycol methacrylate phosphate (EGMP) at different concentrations on PMMA denture resin. They did not highlight statistically significant differences regarding resin bonding ability or other mechanical features. They showed that EGMP concentrations influenced hydrophilicity in a statistically significant way. Faot et al. [54] evaluated accuracy fit, impact strength and performed a fractural analysis on microwave polymerized acrylic resin, using two different polymerizing protocols. Measurements were performed immediately and after 30 days in water storage, showing a better fit for the 30-day group. Tacir et al. [56] in their in vitro study evaluated differences in denture resin mechanical features when reinforced with fiber glass. They showed how fiber glass could improve flexure strength, but also decrease the fracture resistance of a denture in a statistically significant way. Kimoto et al. [57] evaluated dimensional accuracy of heat-cured denture resin with two different cooling protocols, namely, a rapid cooling protocol and a bench cooling. In this last case, the flask was left to cool in a thermo-stabilized room for 140 min. This cooling method provided less denture strain caused by thermal shrinkage. Uzun et al. [59] evaluated the effect of different fiber reinforcement type immediately and after water storage on the strength properties of denture resin. They reinforced the resin with glass and aramid fiber and demonstrated how glass fiber is superior to other fibers and could improve transverse strength. 

Keyf et al. [60] evaluated the effects of hydroxyethyl-methacrylate (HEMA) and air atmosphere on glass fiber, which are used to increase the strength of denture resin. Glass fibers were surface treated with different air and power protocols. HEMA treatments on fiber glass resulted in the modification of the maximal deflection and transverse strength of the denture resin, with no differences in the modulus of elasticity between groups. A study by John et al. [61] evaluated different PMMA fiber reinforcing methods. They highlighted differences on flexural strength of these heat-polymerized resins using glass, aramid and nylon fibers. Glass fibers provided a better performance than the other groups. Test 1 group (glass fiber) had the highest flexural strength, followed by test 2, test 3 and control. The higher the load or force required to fracture the specimens, the higher the fracture resistance.

### 3.6. Additional Analysis

**Figure 3:**
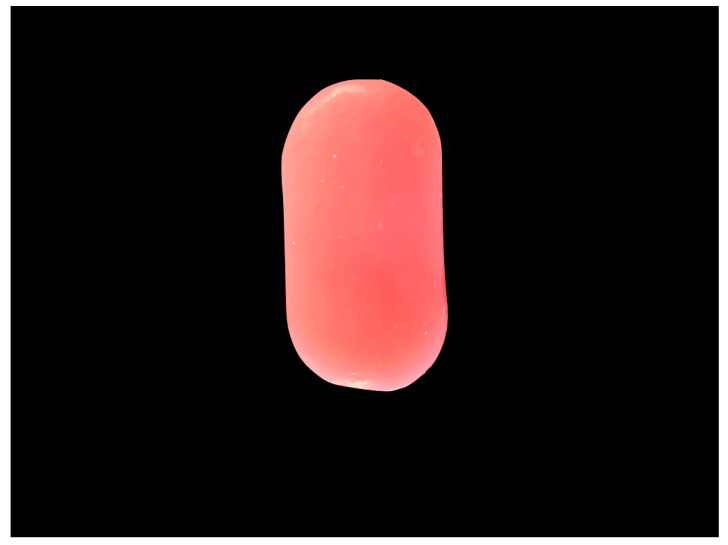
Polished resin sample. It shows a uniform surface with no fibers, cross linking or surface defects.

**Figure 4:**
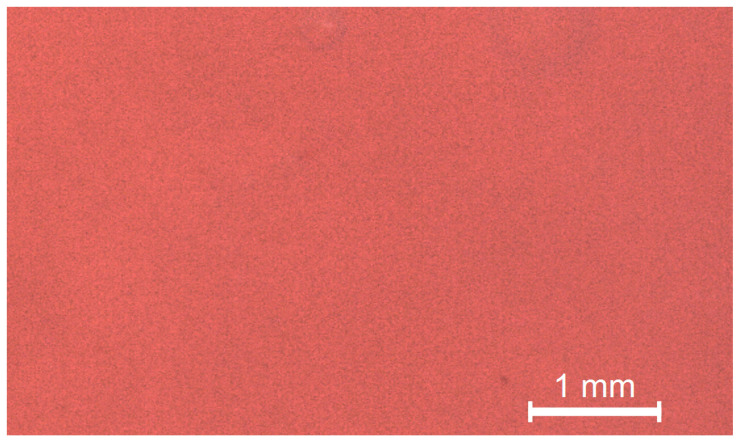
Resin fracture surface observation in stereomicroscopy (Leica^®^ M125 C). It shows fracture lines on a uniform surface.

**Figure 5:**
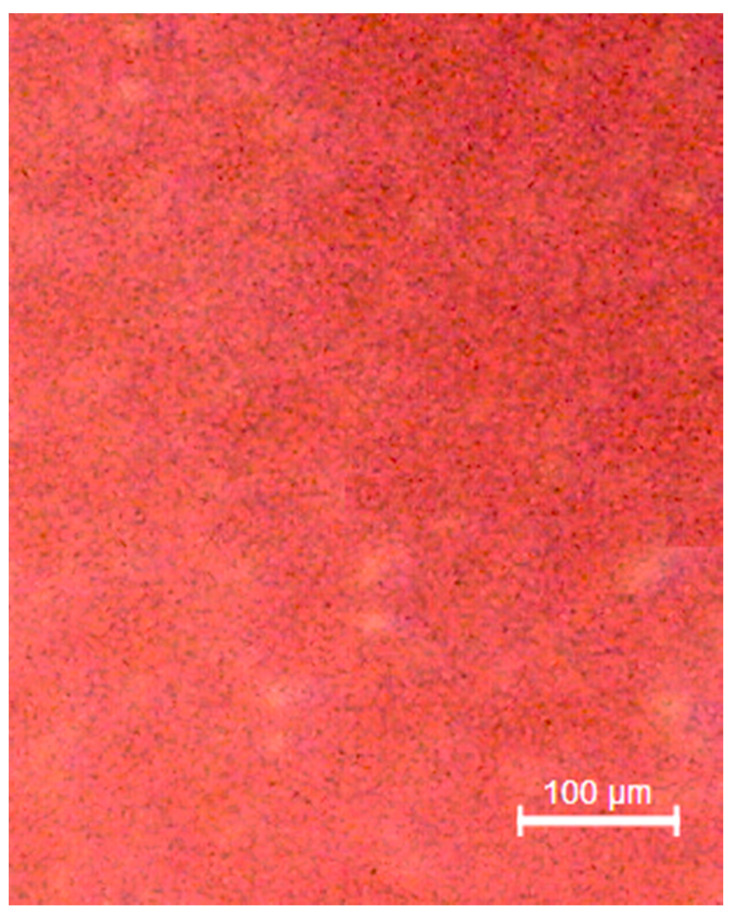
Resin fracture surface observation in stereomicroscopy (Leica^®^ M125 C). It shows a uniform surface.

The data resulting from the tests carried out are visually reported in Figure 2, Figure 3, Figure 4 and Figure 5 in accordance with the Materials and Methods Section.

## 4. Discussion

### 4.1. Summary of Evidence 

#### 4.1.1. Microbiological Properties

According to Bacali et al. [38], PMMA resin loaded with G-AgNp promise good antibacterial properties. Alfaifi et al. [39] affirmed that nicotine and caffeine could affect metabolic activity and biofilm formation of *C. albicans*. In particular, high caffeine concentration could inhibit *C. albicans* metabolism and biofilm formation, but nicotine could increase them on resin. Akalin-Evren et al. [46] evaluated *C. albicans* adhesion on fiber-reinforced dentures with no statistically difference in terms of E-glass FRCs architecture or denture exposition on saliva. According to Fan et al. [51], AgBz-modified resins produced an antibacterial activity against S. mutans. Pesci-Bardon et al. [58] concluded that quaternary ammonium compounds remained active after heat resin polymerization, and that this is a useful aid against Escherichia coli, Staphylococcus aureus and *C. albicans*.

#### 4.1.2. Biological Properties

According to Bacali et al. [38], PMMA resin loaded with G-AgNp caused minimal toxicity to human cells in vitro. Lee et al. [42] evaluated different resin denture materials’ cytotoxicity. Despite the fact all tested materials did not exhibit severe cytotoxicity, potential risk to oral mucosa at high temperatures should not be ignored. The use of 0.5% NaOCl solution for 90 days reduced biofilm formation on dentures [44]. Monteiro et al. [49] evaluated silver dispersion in deionized water at 37 °C, noting that it could affect denture resins with colloidally added antimicrobial silver. They did not detect silver in any specimens. Fan et al. [51], instead, detected Ag+ ion release in all samples. Kim et al. [55] concluded that POSS-reinforced resin had better biocompatibility and less mutagenicity than standard acrylic resins, but 72h of immersion results were similar [62,63,64]. 

#### 4.1.3. Physical Properties

Flexural strength result improved in PMMA resin loaded with G-AgNp [38]. Al-Thobity et al. [40] in their study, showed how different cleansing solutions could modify denture resin properties. In particular some cleansing product could negatively affect color, surface roughness and flexural strength. The authors showed how heat-polymerized, auto-polymerized and visible-light-polymerized react differently. According to Somkuwar et al. [41], heat-polymerized denture base resins with and without reinforcement of MWCNTs and polymerized by the microwave technique possess higher flexural strength. MWCNTs could be used as an effective reinforcement material for the denture base. Wagner et al. [43] demonstrated how the microwaving cycle could affect the dimensional stability of acrylic denture resin. De Sousa Porta [44] did not experienced roughness or color change in acrylic resin dentures after NaOCl solution usage. Wang et al. [45] evaluated the effect of MWCNTs on mechanical features of denture resins. Despite 0.5% and 1 wt% of MWCNTs providing a better flexural strength, this was not the case for the 2 wt% group. According to the authors, it is caused by an inadequate dispersion of carbon nanotubes (that could cause agglomerates in a high percentage) and by the interfacial bonding between MWCNTs and polymethyl methacrylate. According to Mansour et al. [47], the addition of mica addition to PMMA dentures reduced flexural strength, but it significantly increased microhardness. Monteiro et al. [49] showed how antimicrobial denture base resins containing silver colloidal nanoparticles present a better silver distribution and dispersion at a lower silver ratio. So et al. [48] concluded that glass fiber and post-curing microwaving improve the flexural strength in cold-cured PMMA, but it could be influenced by the water storage time of the resin. They suggest a new mixing method called the “sprinkle method” in the fabrication of orthodontic appliances. This involves dispensing the monomers and polymers directly onto the working model. Ladha et al. [50] evaluated how stick and stick net glass fiber-reinforced denture resin and improved resin flexural strength more so than that reinforced with nylon as well as the conventional type. Fan et al. [51] concluded that despite further studies being necessary to evaluate the mechanical properties of AgBz-modified resins, the hardness of chemical-cured resins was not affected. Zortuk et al. [52] concluded that every concentration of fiber glass on acrylic resin affected the surface roughness negatively. Puri et al. [53] concluded that EGMP concentration did not affect the mechanical properties of PMMA dentures, but it did improve hydrophilicity. Faot et al. [54] concluded that different microwave polymerization cycles did not produce different mechanical properties of resin dentures. They showed that after a 30-day storage period in water, the dentures presented a better fit. This result is important because it could indicate a modification process of the denture resin shape. Tacir et al. [56] concluded that glass fiber improve flexural strength of PMMA denture resin, but also decreases fracture resistance. According to the authors, these results could be useful in a clinical setting for the distal extension of partial or total denture bases. Kimoto et al. [57] concluded that bench-controlled cooling produced less thermal shrinkage and reduced strain on the denture. Uzun et al. [59] concluded that glass fiber was superior to other fibers and that it improves transverse strength, deflection and elasticity. Keyf et al. [60] showed how surface treatments and chemical modification on glass fibers could improve strength and maximal deflection of fiber-reinforced denture resin, which could subsequently reduce clinical failures. John et al. [61] in a study approximately 20 years ago guessed and demonstrated that glass fiber provided better results in regard to reinforcing denture resin. According to the authors, glass fiber produced better results than aramid and nylon fibers too.

The stability of the material over time is one of the characteristics that underlies excellent rehabilitation; moreover, the resin should have fracture resistance characteristics and excellent modulus of elasticity [65,66,67,68,69,70]. It has been highlighted how these two characteristics could be influenced by the addition of glass fibers in a significant way, but that there are often inversely proportional characteristics with the use of this material [16,71,72,73]. Another essential feature is represented by bacterial adhesion and bacteriostatic or bactericidal abilities. Many materials have been proposed, with the addition of silver ions appearing to be one of the most valid. Different methods have been suggested for denture cleansing and maintenance, such as modified resin via a cleansing solution or mechanical brushing. Some low-cost methods such as a powered toothbrush could help to maintain and remove biofilm of *C. albicans* on dental acrylic prostheses [74]. Furthermore, the resins should not have cytotoxicity characteristics, and in this field, the addition of polyhedraloligosilsesquioxane has given excellent results [75,76,77,78,79,80,81,82]. The polymeric resinous materials were large stable structures with a high degree of resistance to biodegradation. However, several studies conducted in particular with composite materials have shown that polymers can be subject to degradation processes. In the oral cavity, water is the most abundant component of saliva, as it is one of the main factors causing biodegradation. The oral environment necessarily facilitates the absorption of water from saliva to the resin, which is a polar material. Water molecules can easily penetrate the polymer network, allowing the diffusion of unbound or unpolymerized monomers. Polymeric structures and, in particular, dental materials can also be chemically degraded in aqueous solutions essentially through two mechanisms: hydrolysis and enzymatic reactions [83].

Salivary enzymes can degrade polymers through attacks on the side chains, producing both potential harm to the products and a deterioration of the properties of the network. Water molecules can penetrate the spaces between the polymer chains and further move them away. Consequently, the secondary chemical bonding forces (van der Waals forces) between the polymer chains decrease and correspond to an increase in weight and volume of the material, changing its characteristics. The greater the absorption of water by the material, the greater the dimensional change. The composition of the monomers that produce the network is an important factor in determining the extent of degradation, especially when enzymes are responsible [83].

Interactions between oral microbes and polymer denatured materials may also occur, although little information is available on this possibility. Studies have shown that bacteria can colonize the surfaces of resin-based dental materials. Internal temperature changes can be induced by routine eating and drinking. These temperature changes produce a hostile environment for the materials, as they have a different coefficient of thermal expansion compared to the natural tooth. The thermal fluctuations encountered in vivo can induce surface stress due to the high thermal gradients near the surface. A clinically significant consequence of the biodegradation of acrylic-based resins is the release of potential unbound/uncured monomers and/or additives from the polymer network. Degradation processes not only change the internal properties of the resins, but also affect the bond strength between the prosthetic base resin and the relining material. The compounds released can have a toxic effect on the oral cavity. The biodegradation products of acrylic-based resins have been suspected of being a contributing factor to chemical irritation, sensitization and pain of the oral mucosa. Cell culture techniques have provided strong evidence that compounds released from acrylic-based resins can induce a range of biological responses on cells. The adverse effect mechanism caused by methyl methacrylate monomer (MMA) is believed to involve direct toxicity from released or residual MMA and oxidative stress created by free radicals that are released during polymerization of the resin. In recent years, researchers have used gene expression analysis to evaluate the MMA effect on the expression of antioxidant enzymes such as glutathione. Cell culture techniques have also shown that the residual MMA monomer in acrylic resin-based biomaterials can cause genotoxicity and changes in cytokine/cell growth factor expression [83]. The resins which provide a muffle polymerization, often, despite the issue of volatile substances being debated, allow one to obtain a more stable polymerization of the material. Muffle-free polymerization, on the other hand, often produces a higher quantity of non-polymerized monomer, which affects all the physical and chemical properties of the resin, as already seen in this paragraph [84].

Regarding the physical–mechanical characteristics of a denture, and therefore its clinical predictability, it is necessary to specify that they do not depend only on the materials used, but also on many other factors that could be represented by the design of the prosthesis, the assembly of the teeth, the extension, from patient occlusion or patient parafunctions, or furthermore from poorly managed occlusal loads. Without considering accidental damage (from falling or other), sometimes prostheses, especially if dated, break for no apparent reason, even when eating soft food or drinking hot substances. This may depend on cracks that have formed previously, for example due to a fall, or due to the continuous chewing stresses. This happens especially if the prosthesis does not fit well with the mucosa due to the reabsorption of the underlying bone that occurs over time. Therefore, it is also necessary to keep in mind the maintenance of the dentures. With the periodic relining of the prosthesis, that is, the complete remaking of the surface in contact with the mucosa according to the changes that have occurred in the mouth, these problems will be limited, ensuring a better distribution of forces [78,85].

### 4.2. Limitations 

It is not possible to carry out a meta-analysis or an unequivocal statistical analysis given the large number of results inherent in different and non-comparable analyses, but certainly, even if not numerically, it is possible to draw interesting conclusions on acrylic dental resins. Unfortunately, it is necessary to consider that although some studies taken into consideration the same outcomes, such as flexural strength, the samples and tests were carried out differently.

If in a study a rectangular resin sample has certain dimensions, also in terms of thickness, the resulting parameters will be different. Lamentably there is no uniform way or standard in recreating samples of these materials, therefore, each author chooses the most appropriate method. Unfortunately for this reason, it is not possible to report the data obtained. The same goes for biological or microbiological outcomes where in some cases the parameters taken into consideration are different, while in the last, different microbial species are taken into consideration.

## 5. Conclusions

This review revealed many features of the resins used for dentures, and how much these could be affected by changes to the composition. Some data are essential for the production of new, better performing resins both from a physical, biological and microbiological point of view. In order to create an ideal material, it is appropriate to exploit the individual positive characteristics of these agents, and to create a composite resin with superior properties that could allow one to exploit all these advantages. Surface roughness is one of the factors that definitely influences bacterial adhesion, and this should be reduced as much as possible, as evidenced by the additional study of the resin under consideration. The results of this study provided some important data regarding new resin production: MWCNTs provide better flexural strength at low percentages dispersed in acrylic resins, and glass fibers improve strength and maximal deflection. Bench-controlled cooling of resins should reduce thermal shrinkage and strain on the denture. Silver colloid at a lower ratio could provide an antimicrobial effect for quaternary ammonium compounds in resin. POSS-reinforced resin improve biocompatibility and reduce mutagenicity more so than standard resins. Certainly, other studies are needed, in vitro and in vivo, to bring to light other characteristics of resins and to find the ideal material.

## Figures and Tables

**Table 1:** Study main characteristics and results.

Author and Year	Type of Study	Sample Size and Type	Intervention/Method	Main Outcomes	Main Results	Statistical Analysis
Bacali et al. [38] 2019	Original Article	In vitro study	Auto-polymerizing acrylic resin loaded with 1 wt% (weight) G-AgNp (graphene-Ag nanoparticles) (test1) and 2 wt% G-AgNp (test 2)	Methyl methacrylate monomer (MMA) release; cell (dysplastic oral keratinocytes (DOK) and dental pulp stem cells) viability; oxidative stress and inflammatory response of DOK; antibacterial activity	MMA concentration reached high levels when immersed in chloroform; cell viability displayed a decrease; pro inflammatory molecules (as TNF- α) decreased; antibacterial properties against Gram + bacteria	No significant results of cells viability (*p* = 0.472); significant reduction in TNF- α levels *p* = 0.016 and *p* = 0.104 (for test 1 and 2 respectively); significant antibacterial properties for test 1 and test 2 (*p* < 0.05); significantly improved flexural strength (*P* < 0.05)
Alfaifi et al. [39] 2019	Original Article	240 acrylic resin specimens; in vitro study	Candida albicans metabolic activity (group 1); *C. albicans* biofilm attachment (group 2)	After nicotine and caffeine administration: *C. albicans* metabolic activity; *C. albicans* biofilm attachment	The presence of 8mg/mL of nicotine increased the metabolic activity and biofilm formation of *C*. albicans	Significant *p* < 0.05
Al-Thobity et al. [40] 2019		81 resin specimens; in vitro study	Heat-polymerized (HP) denture base material (group 1); auto-polymerized (AP) denture base material (group 2); visible-light-polymerized (VLP) denture base (group 3)	Denture cleansing solution application, the distilled water group (DWG), Corega group (CG), Renew group (RG): flexural strength; color changes; surface roughness	Color change detected was in the VLP resin treated with Corega and Renew; increased roughness of all denture resin groups after immersion in Corega; reduction in flexural strength in the HP resin after immersion in Corega.	Statistically significant color change *p* < 0.05; significant increase in the surface roughness *p* < 0.05; flexural strength reduction *p* < 0.05
Somkuwar et al. [41]	Original article	180 acrylic resins specimen; in vitro study	Polymethyl methacrylate resin reinforced with 0.025% multiwalled carbon nanotubes (MWCNTs) (group 1); polymethyl methacrylate resin reinforced with 0.050% MWCNTs	conventional water bath groups and microwave group: flexural strength	The mean flexural strength of specimens cured by water bath technique was 95.563 MPa and microwave technique was 118.416 MPa. High percentage of multiwalled carbon nanotubes present better flexural strength	Better flexural strength on microwave group *p* < 0.05; better flexural strength on high MWCNTs groups *p* < 0.05
Lee et al. [42] 2017	Original article	Six thermoplastic resin materials; in vitro study	Three polyamide materials (Smile tone, ST; valplast, VP; and Luciton FRS, LF), two acrylic materials (Acrytone, AT; and Acryshot, AS), and one polypropylene resin material (Unigum, UG), heat-polymerized acrylic resin (Vertex RS, RS) (control)	Extracts and culture with immortalized human oral keratinocytes (IHOKs) or mouse fibroblasts: cytotoxicity	VP at 70° extract and AT at 121° extract showed lower cytotoxicity	*p* < 0.05
Wagner et al. [43] 2017	Original article	20 dentures; in vitro study	PMMA (poly(methyl methacrylate)) acrylic resin	Microwave irradiation at 700W and 420W: dimensional stability	Denture experienced a linear dimensional change of approximately 3%	Significant at *p* < 0.05
De Sousa Porta et al. [44] 2014	Original article	15 participants; clinical study	Acrylic resin dentures	0.5% NaOCl for 3 min over 90 days: biofilm formation, color stability, surface roughness, patient acceptability	Reduction in microorganism and *C. albicans*; no difference in color and roughness; increased level of patient satisfaction	Significant microorganism reduction *p* = 0.001; color *p* = 0.68; roughness *p* = 0.47
Wang et al. [45] 2014	Original Article	In vitro study	Acrylic resin dentures loaded with 0.5, 1, 2 wt% multiwalled carbon nanotubes	Flexural strength	2 wt% MWCNT-loaded dentures showed not beneficial results	Worst mechanical properties on 2 wt% MWCNT-loaded dentures
Akalin-Evren et al. [46] 2014	Original Article	48 denture base resins; in vitro study	Denture base resin reinforced with E-glass fiber-reinforced composites (FRC)	Treated with saliva or distilled water; *C. albicans* adhesion	*C. albicans* adhesion did not show differences	Not significant *p* = 0.436
Mansour et al. [47] 2013	Original Article	199 denture bases; in vitro study	Wet ground muscovite mica and Lucitone 199 original shade denture base resin: (A) control group with 0 vol% mica, (B) 10 vol% W200 mica, (C) 20 vol% W200 mica, (D) 10 vol% P66 mica, (E) 20 vol% P66 mica.	The mica was silane treated in a solution of 3-methacryloxypropyl trimethoxysilane, ethanol, and water, and then dried; flexural strength and microhardness	The flexural strength of the control group 77–94%. No significant differences were found within the four mica groups. Microhardnesses of the 20% mica groups were 33–26%.	Flexural strength higher in control than mica *p* ≤ 0.05. Microhardness of the C group was higher than control (*p* ≤ 0.05).
So et al. [48] 2012	Original Article	50 specimens; in vitro study	Cold cured PMMA with 0%, 2%, 3%, 5% E-glass fibers with and without post-curing microwave at 800 w for 3 min	Water storage for 7,14 and 30 days; Flexural strength, maximum load on the load-deflection curve	The group with 3% fiber and microwave treatment, and the groups with 5% fiber increase in the flexural strength values compared with the control group	Flexural strength on 3% and 5% E-glass fiber(*p* = 0.003 and *p* ≤ 0.003)
Monteiro et al. [49] 2011	Original Article	Denture resin; in vitro study	Denture base resin containing silver colloidal nanoparticles in different concentration 0.05, 0.5, and 5 vol% silver colloidal	Specimens were stored in deionized water at 37 °C for 7, 15, 30, 60 and 120 days; silver distribution and release	Silver was not detected in deionized water; silver distribution and dispersion was improved with lower silver concentration	/
Ladha et al. [50] 2011	Original Article	160 resin specimens; in vitro study	Conventional PMMA denture resin; unidirectional stick (S) glass fiber reinforced-PMMA denture resin; woven stick net (SN) glass fiber-reinforced PMMA denture resin; nylon fiber-reinforced PMMA denture resin	Each group was stored in dry and wet conditions; flexural strength	Glass fiber reinforcements enhanced flexural strength of heat cured PMMA denture	Significant enhanced flexural strength in glass fiber-reinforced group
Fan et al. [51] 2011	Original article	In vitro study	Light-cure denture resins with Ag benzoate of various concentration (0, 0.002, 0.02, 0.1, 0.15 and 0.2%); chemical-cure systems with Ag benzoate various concentration (0, 0.002, 0.02, 0.1, 0.15 and 0.2%)	Resin hardness, silver release, antibacterial activity	Hardness was unaffected by Ag benzoate, and silver was released only at a concentration higher than 0.1%	/
Zortuk et al. [52] 2008	Original article	48 specimens; in vitro study	Auto-polymerizing acrylic resin (no fiber); auto-polymerizing acrylic resin with glass fiber (0.5%); auto-polymerizing acrylic resin with glass fiber (1%); auto-polymerizing acrylic resin with glass fiber (2%)	Surface specimens polishing; surface roughness (Ra)	Difference in resin surface roughness with different concentrations of fiber	*p* < 0.001
Puri et al. [53] 2008	Original article	In vitro study	PMMA resin Lucitone 199; PMMA resin with ethylene glycol methacrylate phosphate (EGMP) 10%; PMMA resin with ethylene glycol methacrylate phosphate (EGMP) 15%; PMMA resin with ethylene glycol methacrylate phosphate (EGMP) 15% + cross linking agent; PMMA resin with ethylene glycol methacrylate phosphate (EGMP) 20%	Impact strength, fracture toughness, wettability, resin bonding ability	Hydrophilicity was increased increasing EGMP concentrations, with no other differences between groups	Improved hydrophilicity *p* = 0.039
Faot et al. [54] 2008	Original Article	In vitro study	Microwave acrylic resin polymerized with 3 min at 360 W, 4-min pause, and 3 min at 810 W (Control); microwave acrylic resin polymerized with an alternative cycle (AC) of 6 min at 630 W	Accuracy of fit at 0 time and at 30 days, impact strength test (Charpy method), fractographic analysis	No difference in outcomes between groups, denture bases showed a better fit after 30-days of storage in water	Better fit after 30 days in water *p* < 0.05
Kim et al. [55] 2007	Original Article	In vitro study	Reinforced acrylic-based hybrid denture composite resin with polyhedraloligosilsesquioxane (POSS) (group 1); heat-polymerized acrylic denture base resin (group 2); auto-polymerized acrylic denture base resin (group 3); direct relining acrylic denture base resin (group 4)	Biocompatibility, mutagenesis	POSS showed improved biocompatibility and lower mutagenicity.	Group 1 showed less cytotoxicity (*p* < 0.05); group 4 showed the highest cytotoxicity (*p* < 0.05)
Tacir et al. [56] 2006	Original article	80 specimens; in vitro study	Conventional heat-polymerized acrylic resin (group 1); Heat-polymerized acrylic resin with glass fibers (10–15μm thick and 5mm long) (group 2); microwaved Shera-Med MW 2000 (Dental-Werkstoffe, Lemförde, Germany) PMMA in a polycarbonate flask (group 3); microwaved Shera-Med MW 2000 (Dental-Werkstoffe, Lemförde, Germany) PMMA in a polycarbonate flask with glass fibers (10–15μm thick and 5mm long) (group 4)	Flexural strength	Group 2 presented better fracture resistance but less flexural strength	*p* < 0.05
Kimoto et al. [57] 2005	Original article	In vitro study	Rapid cooling after heat polymerization (group 1); bench cooling after heath polymerization (group 2)	Denture strain	Bench cooling for the heat-cured denture reduced the strain	*p* < 0.05
Pesci-Bardon et al. [58] 2004	Original article	216 specimens; in vitro study	Acrylic resin discs added with Poly 202063A and large volumes of microbial inoculum (45 mL) (group 1); acrylic resin discs added with Poly 202063A and microbial inoculum (600 microL) (group 2); Acrylic resin discs added with Poly 202063A and sterile buffer (600 microL) (group 3)	Antiseptic properties	A bactericidal effect against Escherichia coli and Staphylococcus aureus. A dose-dependent fungistatic effect was observed against *C. albicans*.	bactericidal effect *p* = 0.012; antifungal effect *p* = 0.003
Uzun et al. [59] 2003	Original article	16 specimens; in vitro study	Pre-treated epoxy resin-coated glass fibers, with aramid fibers, or with no fibers	Immediately and at 30-days water storage; transverse strength, maximal deflection, modulus of elasticity	No differences in strength and deflection values in immediate group and 30 days group	Aramid fiber and without fiber (*p* = 0.574), glass fiber and without fiber (*p* = 0.065) in the immediate group
Keyf et al. [60] 2003	Original article	36 specimens; In vitro study	Auto-polymerizing acrylic resin with hydroxyethyl-methacrylate (HEMA) treated glass fiber: (group A) discharge power of 15 W and flowrate 15 min, 60 mL min; (group B) 20 W, 10 (group C) 15 W, 15 min, 60 mL min)1; (group D) 20 W, 15 min, 60 mL min)1; (group E) untreated; (group F) without fiber	Load of fracture, transverse strength, deflection, modulus of elasticity	Transverse strength and maximal deflection were different between groups, not for modulus of elasticity	Transverse strength *p* = 0.006, deflection *p* = 0.039, elasticity modulus *p* = 0.491
John et al. [61] 2001	Original Article	ten specimens; in vitro study	No fiber reinforced-acrylic resin (control); acrylic resin reinforced with glass fibers (test 1); acrylic resin reinforced with aramid (test 2) acrylic resin reinforced with nylon fibers (test 3)	Flexural strength	All reinforced test groups showed better results on flexural strength; glass fiber showed the highest flexural strength	Test 2 had the best result (*p* < 0.001)

**Table 2:** Risk of bias table according to ROBIS.

Study	Random Sequence Generation	Allocation Concealment	Blinding of Participants and Personnel	Blinding of Outcome Assessment	Incomplete Outcome Data	Selective Reporting	Other Sources of Bias	Overall	Weight
Bacali et al. [38] 2019	High	Low	High	High	Low	Low	Low	Low	/
Alfaifi et al. [39] 2019	High	Low	High	High	Low	Low	Low	Low	240 acrylic resin specimens
Al-Thobity et al. [40] 2019	High	Low	High	High	Low	Low	Low	Low	81 resin specimens
Somkuwar et al. [41] 2017	High	Low	High	High	Low	Low	Low	Low	180 acrylic resin specimens
Lee et al. [42] 2017	High	Low	High	High	Low	Low	Low	Low	Six thermoplastic resin materials
Wagner et al. [43] 2015	High	Low	High	High	Low	Low	Low	Low	20 dentures
De Sousa Porta et al. [44] 2014	High	Low	High	High	Low	Low	Low	Low	15 participants
Wang et al. [45] 2014	High	Low	High	High	Low	Low	Low	Low	/
Akalin-Evren et al. [46] 2014	High	Low	High	High	Low	Low	Low	Low	48 denture base resins
Mansour et al. [47] 2013	High	Low	High	High	Low	Low	Low	Low	/
So et al. [48]	High	Low	High	High	Low	Low	Low	Low	50 specimens
Monteiro et al. [49] 2011	High	Low	High	High	Low	Low	Low	Low	199 denture bases
Ladha et al. [50] 2011	High	Low	High	High	Low	Low	Low	Low	Denture resins
Fan et al. [51] 2011	High	Low	High	High	Low	Low	Low	Low	160 resin specimens
Zortuk et al. [52] 2008	High	Low	High	High	Low	Low	Low	Low	/
Puri et al. [53] 2008	High	Low	High	High	Low	Low	Low	Low	48 specimens
Faot et al. [54] 2008	High	Low	High	High	Low	Low	Low	Low	/
Kim et al. [55] 2007	High	Low	High	High	Low	Low	Low	Low	/
Tacir et al. [56] 2006	High	Low	High	High	Low	Low	Low	Low	/
Kimoto et al. [57] 2005	High	Low	High	High	Low	Low	Low	Low	80 specimens
Pesci-Bardon et al. [58] 2004	High	Low	High	High	Low	Low	Low	Low	/
Uzun et al. [59] 2003	High	Low	High	High	Low	Low	Low	Low	216 specimens
Keyf et al. [60] 2003	High	Low	High	High	Low	Low	Low	Low	16 specimens
John et al. [61] 2001	High	Low	High	High	Low	Low	Low	Low	36 specimens

**Table 3:** Summary of individual outcomes.

Individual Studies Outcomes	
Biological features	Cell viability; oxidative stress and inflammatory response; cytotoxicity; silver distribution and release; biocompatibility, mutagenesis.
Microbiological features	Antibacterial activity; *C. albicans* metabolic activity; C. albicans biofilm attachment; biofilm formation; antiseptic properties.
Physical features	Flexural strength; color changes; surface roughness; dimensional stability; color stability, microhardness; impact strength, fracture toughness, wettability, resin bonding ability; fractographic analysis; denture strain; transverse strength, maximal deflection, modulus of elasticity; load of fracture; maximum load on the load-deflection curve.
Other	Patient acceptability.
